# Impact of depression on quality of life in PKU children

**DOI:** 10.1007/s00431-026-06933-3

**Published:** 2026-04-23

**Authors:** Ibtehal Saad Abuelela, Gihan Mohamed Bebars, Ahmed M. Kamal, Sara Ibrahim Sayed, Rasha Samir Refaat

**Affiliations:** 1 Department of Pediatrics, Faculty of Medicine, Minia University, El-Minia, Egypt; 2 Department of Neuropsychiatry, Faculty of Medicine, Minia University, El-Minia, Egypt; 3 Department of Public Health and Preventive Medicine, Faculty of Medicine, Minia University, El-Minia, Egypt

**Keywords:** PKU, Children, Depression

## Abstract

Phenylketonuria (PKU) is a hereditary metabolic disorder. It is characterized by the accumulation of phenylalanine (Phe) metabolites in the brain and body. These metabolites can harm brain function, especially cognition, if they are not reduced by following a Phe-restricted diet early in life. Despite early treatment with a Phe-restricted diet, many neuro-psychiatric disorders are still reported in adults with PKU. The aim of this study is to assess depression in children with PKU. The study included 76 children aged 8–15 years. They were divided into late-diagnosed, early-diagnosed, and healthy control groups. Participants completed the Birelson depression scale and Peds QL 4.0 to measure health-related quality of life. Phe levels were measured at testing and averaged from the previous year. Children with PKU have higher depression scores than healthy controls. Late-diagnosed children have higher depression scores than early-diagnosed children (*p* = 0.001). Early-diagnosed children differ from controls only in reduced activity (*p* = 0.004). Late-diagnosed children show more loss of energy (85.7%), irritability (78.6%), and suicidal thoughts (21.4%). Quality of life is lower in children with PKU compared to controls, either regarding the total score or all domains. Quality of life is negatively correlated to Phe levels and depression scores (*r* =  − 0.32 and *r* =  − 0.51, respectively) while positively correlated to IQ levels (*r* = 0.74).

*Conclusion*: Children with PKU show more depression and lower quality of life. These effects may be linked to diagnosis timing, long term Phe control, and IQ. Depression is more pronounced in late-diagnosed children than in early-diagnosed ones.

**What is Known:**

• *Patients with PKU are at increased risk of psychiatric disorders.*

• *Poor metabolic control (elevated Phe levels) is associated with lower IQ and QoL in PKU patients.*

**What is New:**

• *Children with PKU have higher levels of depressive symptoms, especially in late-diagnosed children, who develophigher rates of suicidal thoughts.*

• *Diagnosis timing, phe levels, depression severity, and IQ together strongly predict QoL (explaining up to 61% ofvariance).*

## Introduction

Phenylketonuria (PKU) is relatively common in Egypt, with an incidence ranging from 1 in 2638 to 1 in 3000 live births according to the Egyptian National Newborn Screening Program (2018–2021) [1].

PKU is an inherited metabolic disorder caused by mutations in the phenylalanine hydroxylase (PAH) gene, leading to a deficiency of the enzyme responsible for converting Phe to tyrosine [2]. Elevated Phe levels disrupt neurotransmitter synthesis and brain amino acid balance, resulting in neurological and psychiatric impairment [3].

Phenylketonuria due to PAH deficiency ranges from classical PKU (untreated blood Phe > 1200 µmol/L, indicating complete enzyme deficiency) to milder forms. However, the National Institutes of Health classifies patients with elevated but < 1200 µmol/L Phe as hyperphenylalaninemia and recommends unified terminology for the full PAH deficiency spectrum [4]. The severity of neuropsychiatric outcomes varies accordingly; untreated classical PKU leads to severe intellectual disability, whereas milder forms carry a lower risk [2, 4].

Early diagnosis through newborn screening (NBS) is essential to prevent long-term complications. Most countries have implemented NBS programs, enabling early dietary treatment and markedly reducing severe clinical manifestations [3, 5]. In Egypt, NBS for PKU was introduced in November 2015.

Major depressive disorder (MDD) is estimated to affect approximately 2% of children and 4–8% of adolescents in community-based (general population) samples in North America. The male-to-female ratio is approximately 1:1 in childhood and shifts to about 1:2 during adolescence, with higher prevalence observed in females [6, 7]

Although pediatric data in PKU are limited, studies in adults suggest higher rates than in the general population [8]. Additionally, lifelong dietary restrictions, frequent blood monitoring, and Repeated medical visits may negatively affect HRQoL [9]. Therefore, this study aims to assess the prevalence and severity of depressive symptoms and to evaluate HRQoL in early- and late-treated children and adolescents with PKU, as well as to examine the association between at the test time and long-term blood Phe levels and the psychological outcomes.

## Patients and methods

### Study design and participants

This is a descriptive cross-sectional case–control study. The sample size was 76 subjects; 50 children diagnosed as PKU (divided into 2 groups: late-diagnosed group, 28 cases, and early-diagnosed group, 22 cases) and 26 age and sex matched controls.

The inclusion criteria were (a) children with classical PKU on a phenylalanine-restricted diet, (b) early- and late-diagnosed PKU children aged from 8 to 15 years, and (c) only one affected child per family (no PKU siblings) to be included in the study. The exclusion criteria included the following: (a) children with comorbid neurological and\or other psychiatric illness, like epilepsy, attention deficit hyperactivity disorder, autism spectrum disorder; (b) children who were diagnosed with benign mild HPA (initial phe levels at diagnosis were 120 to 360 micromol/L) and does not require therapy or diet restriction and those with BH4 deficiency; and (c) children with IQ lower than 50.

Cases were recruited from the clinic of inborn errors of metabolism at the Children’s Hospital of Minia University, Minia Governorate, during the period from May to December 2023. The control group was randomly selected from non-PKU healthy children, age and sex matched.

### Ethical consideration

The study received Institutional Review Board approval (Faculty of Medicine, Minia University; No. 699:2023; 13 March 2023), and written informed consent was obtained from caregivers.

### Data collection

A structured interview with parents/guardians collected socio-demographic and disease-related data (age at diagnosis, family history, affected siblings, consanguinity, residence, and maternal education). All children underwent general and neurological examinations to identify associated medical or neurological conditions. Standardized, validated instruments were used to assess depressive symptoms and HRQoL, administered according to guideline recommendations with assistance provided when necessary.

### Tools of the study

#### Birleson depression self-rating scale for children (DSRS-C) [10]

The DSRS-C is an 18-item self-report scale for children aged 8–14 years, assessing depressive symptoms including mood, anhedonia, sleep disturbances, cognitive, and somatic changes. Each item is rated on a 3-point scale (1 = never, 2 = occasionally, 3 = frequently), and total scores ≥ 17 may indicate clinically significant depression.

#### Pediatric quality of life (PedsQL 4.0) [11]

HRQoL was assessed using the Arabic version of the PedsQL 4.0, a 23-item questionnaire covering four domains: physical (8 items), emotional (5 items), social (5 items), and school functioning (5 items). Items are rated on a 5-point Likert scale (0 = never a problem to 4 = almost always a problem). Raw scores for each subscale were averaged and transformed to a 0–100 scale (Transformed score = 100 − [Mean raw score × 25]). The resulting score is a number between 0 and 100, where: Higher scores indicate better quality of life, with scores ranging from 80 to 100 representing excellent to good functioning, 60–79 fair, 40–59 poor, and below 40 very poor. In this study, results are reported as separate domain scores and as two composite scores: Physical Health and Psychosocial Health (average of emotional, social, and school domains), as well as the total score (mean of all items) [12, 13].

#### Stanford-binet intelligence scale, fifth edition [14]

To measure the intelligence quotient (IQ) Stanford-Binet Intelligence Scale, 5th edition, was used by an expert psychologist.

#### Phenylalanine measurement

Patients were monitored according to the 2017 European PKU guidelines, targeting phenylalanine levels of 120–360 µmol/L (< 12 years) and 120–600 µmol/L (≥ 12 years). Follow-up was conducted every three months with dried blood spot analysis using the NeoBase Non-derivatized kit at the Central Health Laboratories. Phenylalanine measured on the day of psychological assessment was defined as “Phe at test time,” while the median value over the preceding year was calculated to reflect long-term metabolic control.

### Data analysis

Data were analyzed using SPSS version 25. Normality was assessed with the Kolmogorov–Smirnov test. Parametric data were expressed as mean ± SD and compared using independent t-tests or one-way ANOVA with Bonferroni post hoc tests. Non-parametric data were presented as median (IQR) and analyzed using Kruskal–Wallis and Mann–Whitney *U* tests. Categorical variables were summarized as frequencies and percentages and compared using Chi-square or Fisher’s exact tests. Spearman correlation assessed associations between depression scores, QoL, and phenylalanine levels. Hierarchical linear regression was used to identify independent predictors of QoL, adjusting for potential confounders (residence, maternal education, IQ). Statistical significance was set at *p* < 0.05.

## Results

Demographic differences among the studied groups were illustrated in Table 1, Rural residence and illiterate maternal education were more prevalent among late-diagnosed patients (100%, 50%) respectively.

**Table 1:** Demographic characteristics for studied groups

Variable	Early-diagnosed cases (*n* = 22)	Late-diagnosed cases (*n* = 28)	Controls (*n* = 26)	*p* value (overall)	P1	P2	P3
Age (years)							
Median (Q1–Q3)	8 (8–9)	10 (9–15)	10 (8–11)	0.001*	< 0.001*	0.022*	0.213
Sex							
Male	8 (36.4%)	10 (35.7%)	14 (53.8%)	0.327	0.962	0.226	0.379
Female	14 (63.6%)	18 (64.3%)	12 (46.2%)				
Consanguinity							
Negative	4 (18.2%)	2 (7.1%)	26 (100%)	0.001*	0.385	< 0.001*	< 0.001*
Positive	18 (81.8%)	26 (92.9%)	0 (0%)				
Residence							
Rural	16 (72.7%)	28 (100%)	10 (38.5%)	0.001*	0.003*	0.011*	< 0.001*
Urban	6 (27.3%)	0 (0%)	16 (61.5%)				
Maternal education							
Illiterate	8 (36.4%)	14 (50%)	4 (15.4%)	0.001*	0.022*	< 0.001*	< 0.001*
Primary educated	0 (0%)	6 (21.4%)	0 (0%)				
Preparatory	2 (9.1%)	2 (7.1%)	6 (23.1%)				
Secondary	12 (54.5%)	6 (21.4%)	4 (15.4%)				
University	0 (0%)	0 (0%)	12 (46.2%)				

- Kruskal–Wallis test used to compare quantitative data between three groups, Mann–Whitney test was used for pairwise comparison

- Chi-square/Fisher exact test was used to compare qualitative data between three groups

^*^Significant difference (*p* value ≤ 0.05) between three groups

P1: *p* value between early and late-diagnosed groups

P2: *p* value between early-diagnosed and control groups

P3:*p* value between late-diagnosed and control groups

At the test time and median phenylalanine levels did not differ significantly (*p* > 0.05) between the two PKU groups. Regarding the number of affected siblings, no significant differences were observed between the two groups (Table 2).

**Table 2:** Clinical characteristics for studied groups

Variable	Early-diagnosed cases (*n* = 22)	Late-diagnosed cases (*n* = 28)	*p* value
Age of diagnosis			
Median (Q1–Q3)	0 (0–0)	6.25 (2–8)	< 0.001*
Family history			
Negative	10 (45.5%)	8 (28.6%)	0.217
Positive	12 (54.5%)	20 (71.4%)	
Number of affected siblings			
1	12 (54.5%)	16 (57.1%)	0.271
2	0 (0%)	4 (14.3%)	
At the test time phenylalanine level			
Median (Q1–Q3) (µmol/L)	480 (294–650)	426 (200–564)	0.584
Median phenylalanine level			
Mean ± SD (µmol/L)	473.9 ± 198	534.4 ± 300	0.268

- Mann − Whitney *U* test used to compare nonparametric quantitative data between two groups

- Independent sample *t* test to compare parametric quantitative data between two groups

- Chi-square test/Fisher exact test was used to compare qualitative data between three groups

^*^Significant difference (*p* value ≤ 0.05)between all PKU subjects (early- and late-diagnosed) and controls

Total depression score, quality of life (QoL), and IQ are summarized in Table 3. Median depression scores were 3 (IQR 1–7) for early-diagnosed PKU, 7 (IQR 5–9) for late-diagnosed PKU, and 2 (IQR 0–5) for controls (*p* = 0.001), with higher depressive symptoms observed in late-diagnosed cases; 14.3% of late-diagnosed patients scored 11–17, compared to none in early-diagnosed patients or controls. QoL was lower in late-diagnosed patients, particularly in school and psychosocial domains, with total scores significantly lower than those of early-diagnosed cases and controls. IQ was also markedly lower in late-diagnosed patients compared to early-diagnosed patients and controls (*p* < 0.001).

**Table 3:** Depression score, quality of life and IQ for studied groups

Variable	Early-diagnosed cases **(*n* = 22)**	Late-diagnosed cases **(*n* = 28)**	Controls **(*n* = 26)**	*p* value (overall)	P1	**P2**	**P3**
Total Birleson depression scale							
Median (Q1–Q3) < 11 11–17	3 (1–7) 22 (100%) 0 (0%)	7 (5–9) 24 (85.7%) 4 (14.3%)	2 (0–5) 26 (100%) 0 (0%)	0.001* 0.033*	0.001* 0.121	0.769 1	< 0.001* 0.112
- Sleep disturbance - Appetite disturbance - Loss energy - Increase irritability - Reduce activity - Expressed self-deprecating ideas - Suicidal thread - New somatic complain - Wander behavior - Depression delusion	6 (27.3%) 2 (9.1%) 10 (45.5%) 10 (45.5%) 6 (27.3%) 0 (0%) 0 (0%) 6 (27.3%) 2 (9.1%) 8 (36.4%)	10 (35.7%) 6 (21.4%) 24 (85.7%) 22 (78.6%) 10 (35.7%) 6 (21.4%) 6 (21.4%) 18 (64.3%) 10 (35.7%) 20 (71.4%)	4 (15.4%) 2 (7.7%) 8 (30.8%) 8 (30.8%) 0 (0%) 4 (15.4%) 0 (0%) 10 (38.5%) 4 (15.4%) 6 (23.1%)	0.236 0.331 0.001* 0.002* 0.004* 0.058 0.003* 0.024* 0.049* 0.001*	0.525 0.439 0.002* 0.015* 0.525 0.028* 0.028* 0.009* 0.029* 0.013*	0.312 1 0.375 0.295 0.006* 0.055 1 0.413 0.674 0.313	0.089 0.253 < 0.001* < 0.001* < 0.001* 0.730 0.024* 0.058 0.089 < 0.001*
Quality of life score							
- Physical - Psychological - Social - School - Psychosocial - Total score	100 (84.3–100) 85 (70–90) 95 (80–100) 90 (85–100) 90 (73.3–95) 91.7 (72.9–96.2)	86.1 (81.2–93.4) 80 (75–90) 72.5 (65–90) 50 (45–85) 73.3 (63.3–78.3) 72.3 (68.2–80)	100 (93.7–100) 95 (95–100) 100 (95–100) 100 (93.7–100) 96.6 (92.5–100) 97.5 (93.2–100)	0.001* < 0.001* 0.001* < 0.001* 0.001* < 0.001*	0.004* 0.302 < 0.001* 0.001* 0.001* < 0.001*	0.287 < 0.001 0.020* 0.012* < 0.001* 0.001*	< 0.001* < 0.001* < 0.001* < 0.001* < 0.001* < 0.001*
IQ	94 ± 8.6	65.8 ± 9.3	104.2 ± 12.9	< 0.001*	< 0.001*	0.004*	< 0.001*

- Quantitative data were presented by mean ± SD, median (IQR), while qualitative data were presented by number and percentage

- Kruskal–Wallis test used to compare nonparametric quantitative data between three groups

- Mann–Whitney *U* test used to compare nonparametric groups quantitative data between two groups

- One way, the ANOVA test is used to compare parametric quantitative data between three groups

- Chi-square test was/Fisher exact test used to compare qualitative data between three groups

^*^Significant difference (*p* value ≤ 0.05) between three groups

P1: *p* value between early and late-diagnosed groups

P2: *p* value between early-diagnosed and control groups

P3:*p* value between late-diagnosed and control groups

Total depression scale was negatively correlated with the total quality of life sore and IQ levels (*r* =  − 0.51, *p* < 0.001), indicating that higher depressive symptoms were associated with poorer QoL. However, total quality of life score was positively correlated with IQ levels (*r* = 0.74, *p* < 0.001), suggesting that better cognitive functioning is associated with improved quality of life (Table 4).

**Table 4:** Correlation between phenylalanine level and quality of life scores and total depression scores for cases (*n* = 50)

Variable #	1	2	3	4	5	6	7	8	9
1. Median phenylalanine level	1								
2. Physical	− 0.30*	1							
3. Psychological	− 0.22	0.27	1						
4. Social	− 0.38**	0.45**	0.35*	1					
5. School	− 0.30*	0.61**	0.52**	0.51**	1				
6. Psychosocial	− 0.27	0.63**	0.68**	0.77**	0.84**	1			
7.Total score	− 0.32*	0.75**	0.67**	0.71**	0.88*	0.96**	1		
8. IQ	− 0.22	0.63**	0.23	0.61**	0.75**	0.67**	0.74**	1	
9. Birleson depression scale	0.40**	− 0.47**	− 0.15	− 0.51**	− 0.48**	− 0.39**	− 0.51**	− 0.61**	1

^#^Spearman correlation was calculated to define the strength and direction of the linear relationship between two variables, denoted symbolically r: Grades of r: 0.00 to 0.24 (weak or no correlation), 0.25 to 0.49 (fair correlation), 0.50 to 0.74 (moderate correlation), > 0.75 (strong correlation)

^*^p value < 0.05 (two-tailed)

^**^p value < 0.01 (two-tailed)

Hierarchical regression examined the effects of phenylalanine level, delayed diagnosis, socioeconomic factors, depression, and IQ on quality of life (QoL) in PKU patients. Model 1 showed that delayed diagnosis significantly predicted lower QoL (*β* = –1.18) and explained 35% of the variance, independent of socioeconomic factors. Adding depression score in Model 2 increased the explained variance to 44% (*B* = –1.11, *p* = 0.012), and inclusion of IQ in Model 3 further raised it to 61% (*p* < 0.001), indicating that cognitive function substantially influences QoL outcomes (Table 5).

**Table 5:** Hierarchical linear regression analysis for factors predicting quality of life for cases (*n* = 50)

Independent variable	Model 3 B	Model 2 B	Model 1 B
Median phenylalanine level	− 0.014*	− 0.014*	− 0.011
Onset of diagnosis	0.213	− 0.673	− 1.18*
Residence	− 0.284	5.19	9.15
Maternal education	− 1.010	0.441	0.392
Total depression score	− 0.386	− 1.11*	-
IQ	0.502*	-	-
Model summary			
Model significance * R*^2^ Adjusted *R*^2^ * R*^2^ change	< 0.001* 0.613 0.557 0.172	0.012* 0.442 0.375 0.092	< 0.001 0.350 0.290 -

^*^p value < 0.05

## Discussion

Our study shows significantly higher depressive symptoms in children with PKU compared to controls, suggesting an increased risk of depression. This agrees with Pietz et al. [8], who reported higher rates of psychiatric disorders, especially depression, in adults with PKU. High Phe levels may reduce tyrosine, catecholamines, and serotonin [15], and the brain damage from Phe accumulation may contribute to depression [16].

Depressive symptoms are more severe in late-diagnosed than early-diagnosed cases. This suggests that prolonged Phe exposure and delayed diagnosis worsen outcomes. These findings are consistent with Sharman et al. [16], who linked depressive symptoms to long-term high Phe: tyrosine ratios or low tyrosine levels in adolescents. A recent meta-analysis also reported depression in 12% of early-treated adults versus 35% of late-treated adults [17].

Coming from rural areas, illiterate mothers may also have an impact on the severity of depressive symptoms. All late-diagnosed children live in rural areas, and half of their mothers are illiterate. These factors are less evident in the early-diagnosed group. These factors may delay diagnosis, complicate treatment, and consequently increase depressive symptoms due to limited healthcare access, financial problems, and poor disease awareness [18]. All these mentioned factors may contribute to suboptimal metabolic control observed in our PKU sample (early or late-diagnosed groups).

Early-diagnosed cases show significantly reduced activity (27.3%) compared to the control group. This finding indicates the presence of subtle cognitive and behavioral deficits despite early treatment, which is supported by Crossley and Anderson’s study [19].

Late-diagnosed cases show significantly more depressive symptoms than both early-diagnosed and control groups. Loss of energy (85.7%) and irritability (78.6%) are the most common symptoms, consistent with previous studies [20–22]. They also show more psychosis, reduced activity, and wandering behavior, possibly due to lower IQ and brain injury from prolonged high Phe levels [23].

Suicidal thoughts are reported in 21.4% of late-diagnosed cases, significantly higher than in other groups. This may be related to severe depression, lower IQ, low self-esteem [23], poor quality of life, and social difficulties.

IQ is significantly lower in PKU cases than in controls, especially in late-diagnosed children. Early treatment (before 1–2 months) prevents severe intellectual disability seen in late or untreated cases [24–26]. However, even early treated children often have lower IQ than peers or family members [27]. Netley et al. [24] reported IQ scores more than 10 points below parental levels.

PKU patients, both early- and late-diagnosed, have significantly lower quality of life (QoL) than controls. This agrees with Vieira et al. [28] results in Brazilian children. Lower QoL may result from the chronic nature of PKU and continuous treatment demands. However, some studies report good health-related QoL in PKU patients of all ages [29, 30]. Unlike those studies, ours compared PKU children with healthy matched controls, which may explain the difference.

Late-diagnosed children have worse QoL than early-diagnosed cases. The same finding was reported by Hatami et al. [31]. Late diagnosis, Lower IQ, more severe depressive symptoms, and, to a lesser extent, rural residence and maternal illiteracy may explain this finding.

QoL is negatively correlated with median Phe levels (*r* =  − 0.36) and total depression score (*r* =  − 0.51) and positively correlated with IQ (*r* = 0.74). Similar findings were reported by many researchers linking poor metabolic control (as in our sample) to lower QoL [9, 29, 32].

No previous study has specifically examined QoL in PKU for children with depression. However, Celebre et al. [33] reported that in the general population of children and adolescents, the presence of depressive symptoms reduces social and personal QoL. Kul et al. [34] showed that chronically ill children, such as those with chronic kidney disease, have poorer QoL than healthy controls. Thus, children with both PKU and depression may experience even lower QoL.

Quality of life (QoL) is positively correlated with IQ. Higher IQ may improve access to education, employment, income, and housing, which are associated with better QoL [35].

Hierarchical regression shows that diagnosis timing and Phe levels explain 35% of QoL variance. Adding depression score and IQ increases the explained variance to 61%, highlighting the impact of IQ and depression on QoL in PKU children. However, it is difficult to look at complications separately, as elevated blood Phe leads to lower IQ and a greater incidence of depression.

This study has both strengths and limitations. The strength lies in examining depressive symptoms and their effect on quality of life in children with PKU, a relatively understudied area. It also identifies key factors influencing QoL, including depression, IQ, Phe levels, maternal education, and place of residence. The limitations are that while Peds QL 4.0 was used to allow comparison with healthy controls, the lack of a PKU-specific QoL tool may have limited assessment of disease-related burden. Additional limitations include a small sample size, single-center design, suboptimal metabolic control in PKU cases (early- or late-diagnosed), and the use of a self-reported depression scale.

## Conclusion and recommendation

PKU negatively affects mood, IQ, and quality of life, especially in late-diagnosed cases. Early detection through newborn screening and proper treatment improves outcomes. Monitoring depressive symptoms is essential to prevent more serious mental health issues.

## Data Availability

The data used and/or analyzed during the current study are available from the corresponding author on a reasonable request.

## Abbreviations

DSRS-C: Birleson depression self-rating scale for children
HRQoL: Health-related quality of life
IQ: Intelligence quotient
NBS: Newborn screening
PAH: Phenylalanine hydroxylase
Peds QL 4.0: Pediatric quality of life
Phe: Phenylalanine
PKU: Phenylketonuria
QoL: Quality of life
